# MicroRNA-92b promotes hepatocellular carcinoma progression by targeting Smad7 and is mediated by long non-coding RNA XIST

**DOI:** 10.1038/cddis.2016.100

**Published:** 2016-04-21

**Authors:** L K Zhuang, Y T Yang, X Ma, B Han, Z S Wang, Q Y Zhao, L Q Wu, Z Q Qu

**Affiliations:** 1 Department of Hepatobiliary and Pancreatic Surgery, The Affiliated Hospital of Qingdao University, Qingdao 266003, China; 2 Center for Medical Research, The Affiliated Hospital of Qingdao University, Qingdao 266003, China

## Abstract

MicroRNA (miRNA) and long non-coding RNA (lncRNA) have been demonstrated to participate in the progression of many cancers. Hepatocellular carcinoma (HCC) is one of the most common and aggressive malignant tumors worldwide, while the molecular mechanisms underlying HCC tumorigenesis are not completely clear. In this study, we showed that miR-92b was significantly upregulated in tumor tissue and plasma of HCC patients, and its expression level was highly correlated with gender and microvascular invasion. Functionally, miR-92b could promote cell proliferation and metastasis of HCC *in vitro* and *in vivo*. Mechanistic investigations suggested that Smad7, which exhibited an inverse relationship with miR-92b expression in HCC, was a direct target of miR-92b and could reverse its effects on HCC tumorigenesis. Furthermore, long non-coding RNA (lncRNA) X-inactive specific transcript (XIST) and miR-92b could directly interact with and repress each other, and XIST could inhibit HCC cell proliferation and metastasis by targeting miR-92b. Taken together, our study not only revealed for the first time the importance of XIST/miR-92b/Smad7 signaling axis in HCC progression but also suggested the potential value of miR-92b as a biomarker in the clinical diagnosis and treatment of HCC.

Accounting for about 90% of liver tumors, hepatocellular carcinoma (HCC) is one of the most common human cancers in the world with high mortality and poor prognosis.^1^ In recent years, liver resection, liver transplantation and radiofrequency ablation are common treatments for HCC. However, owing to the recurrence and metastasis, the overall survival for HCC patients remains unsatisfied.^2^ To reduce recurrence and raise survival rate, the in-depth exploration of molecular mechanism underlying HCC tumorigenesis and the further search for new molecular targets affecting tumor growth and metastasis are required in both clinical diagnosis and treatment for patients with HCC.

In the past few years, non-coding RNAs including microRNA (miRNA) and long non-coding RNA (lncRNA) have been widely reported as a new class of clinical biomarkers and potential therapeutic targets for cancers.^3, 4^ As highly conserved small non-coding RNAs with sizes of 20–25 nucleotides, miRNAs could negatively regulate gene expression at post-transcriptional level by directly binding to the 3′-untranslated region (3′-UTR) of target messenger RNA (mRNA) to induce mRNA deregulation or translational repression.^5^ Many miRNAs have been demonstrated to participate in the pathogenesis of HCC as oncogenes or tumor suppressor genes, which could target cell signaling pathways to control various biological processes including cell proliferation, apoptosis, differentiation and migration.^6, 7^ For instance, miR-188-5p, which was downregulated in HCC, could target FGF5 to affect MEPK/ERK pathway and finally inhibit tumor cell proliferation and metastasis.^8^ Although more and more miRNAs have been demonstrated as important regulators in HCC pathogenesis, the relationship between the majority of miRNAs and HCC remains to be explored.

In recent years, lncRNA has been demonstrated to be a sponge for regulating the expression and activity of miRNA.^9, 10^ LncRNAs with a size >200 nucleotides could have an important role in the regulation of gene expression during the pathogenesis of diseases including cancers by the mechanisms that are not yet fully understood. Moreover, more and more experimental evidences suggested that one potential function of lncRNA was to interact with miRNA and regulate its expression and activity. For example, the lncRNA X-inactive specific transcript (XIST) and miR-152 could interact with and repress each other, finally acting as crucial regulators for function of human glioblastoma stem cells.^11^ However, limited knowledge is available concerning the relationship between lncRNA and miRNA in the carcinogenesis of HCC, which needs to be well documented.

In the present study, for the first time we reported that miR-92b was upregulated in tumor tissue and plasma of HCC patients, which in turn promoted the proliferation and metastasis of HCC cells *in vitro* and *in vivo*. Then, we investigated the molecular mechanism of miR-92b in the progression of HCC and the reciprocal regulation between miR-92b and lncRNA XIST. Our findings provided new insights into the molecular function of XIST/miR-92b/Smad7 signaling pathway in HCC tumorigenesis and suggested the potential value of miR-92b in the clinical diagnosis and treatment of HCC.

## Results

### MiR-92b is upregulated in tumor tissue and plasma of HCC

**Figure 1:**
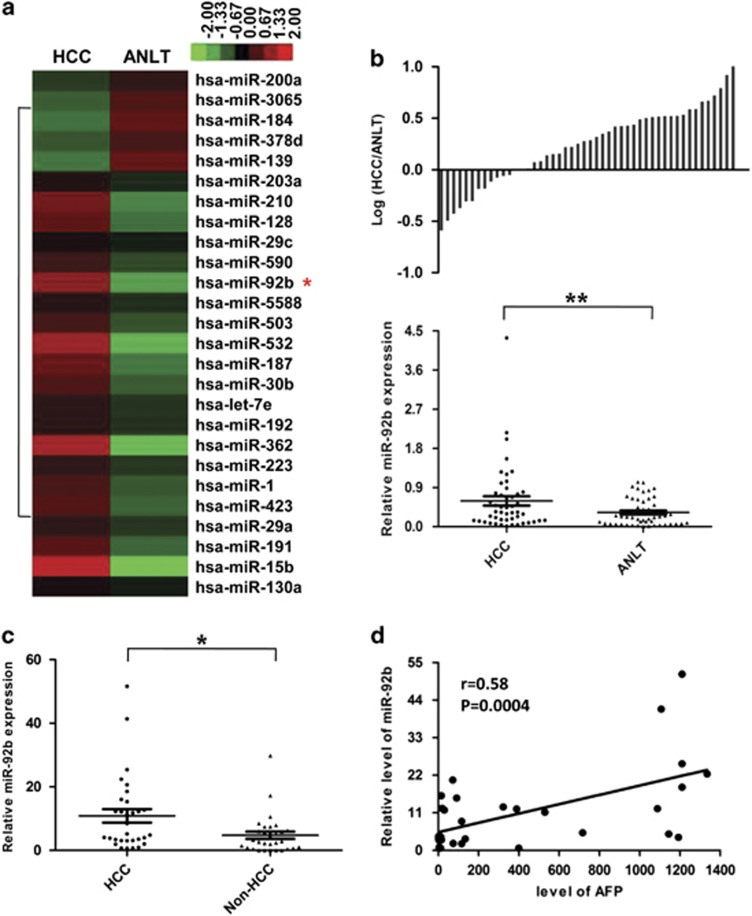
Expression levels of miR-92b in HCC tissues and plasma samples. (**a**) Partial miRNAs expression profiles of HCC tissues and ANLTs. Red or green color in heatmap separately indicates high or low expression, according to the color bar in logarithmic scale shown above the heatmap. (**b**) Expression levels of miR-92b in 48 pairs of HCC tissues and ANLTs were determined by qRT-PCR and normalized to U6 expression. The data were analyzed by delta Ct method. Upper panel: The bars represent the relative miR-92b expression with the ratio of its level in HCC tissue *versus* ANLT in logarithmic scale. Lower panel: The miR-92b expression levels in HCC tissues and ANLTs were compared with paired Student's *t*-test. (**c**) miR-92b levels in the plasma of HCC patients (*n*=31) and patients without liver diseases (*n*=29). The expression levels were compared with unpaired Student's *t*-test. (**d**) The positive correlation between AFP and miR-92b levels in the plasma of HCC patients (*r*=0.58, *P*=0.0004, *n*=31). *P<0.05; **P<0.01

To explore the expression and significance of miRNAs in HCC, small RNA high-throughput sequencing was performed to compare the miRNA expression patterns between HCC and paired adjacent non-tumor liver tissues (ANLTs). We found that miR-92b, which could act as an oncogene in many cancers but was rarely reported in HCC, was one of the upregulated miRNAs in HCC tissues compared with ANLTs (Figure 1a). Then, we measured miR-92b levels in 48 pairs of HCC tissues and ANLTs by quantitative reverse transcription-PCR (qRT-PCR). Consistent with small RNA sequencing data, the expression level of miR-92b in HCC tissues was significantly higher than that in ANLTs (Figure 1b).

To explore the correlation between miR-92b and clinicopathological variables, miR-92b expressions in HCC tissues were divided into two groups including the high-expression group (higher than that in corresponding ANLT) and the low-expression group (lower than that in corresponding ANLT). Then, the correlations of miR-92b expression with clinicopathologic characteristics of HCC were analyzed. The results showed that miR-92b expression level was relatively higher in HCC tissues of female patients or with microvascular invasion (Table 1).

We also attempted to detect the level of circulating plasma miR-92b in patients with HCC. Interestingly, compared with patients without liver diseases, HCC patients showed a significantly higher level of plasma miR-92b expression (Figure 1c). As circulating *α*-fetoprotein (AFP) is frequently used for the diagnosis of HCC, we also examined the relationship between AFP and miR-92b in the plasma and found that the expression of miR-92b in the plasma of HCC patients was positively associated with the corresponding AFP level (Figure 1d). The correlation indicated that miR-92b might be a potential biomarker in the clinical diagnosis of HCC.

### MiR-92b promotes HCC cell proliferation and metastasis *in vitro*

**Figure 2:**
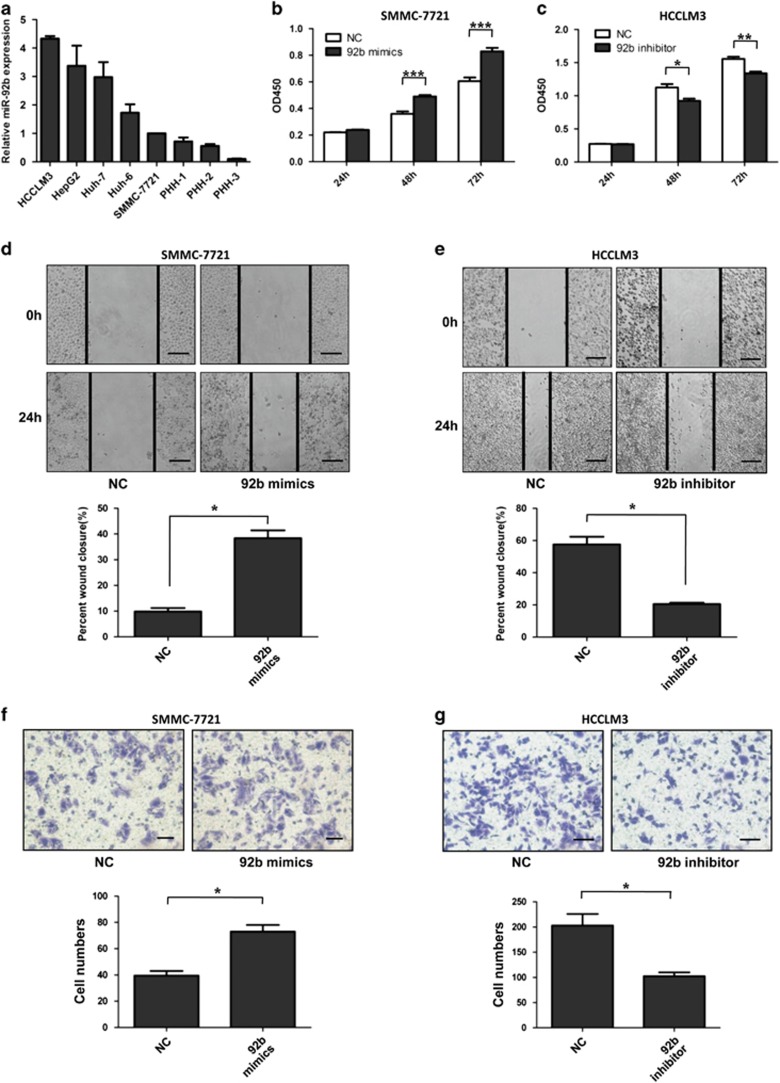
Effects of miR-92b on HCC cell proliferation and metastasis *in vitro*. (**a**) The relative miR-92b expression in five HCC cell lines (SMMC-7721, Huh-6, Huh-7, HepG2 and HCCLM3) and three primary human hepatocytes (PHH-1, 2 and 3) were determined with qRT-PCR. The data were normalized to the expression level of miR-92b in SMMC-7721 cells. Results were represented as mean±S.D. (*n*=3). (**b**–**g**) miR-92b was overexpressed in SMMC-7721 cells while knocked down in HCCLM3 cells. CCK-8 analysis (**b** and **c**), the wound healing assays (**d** and **e**) and transwell invasion assays (**f** and **g**) were conducted. The percent of wound closure and the number of cells passed through the membrane was counted and compared in the diagrams. Scale bars represent 500 *μ*m (**d** and **e**) and 200 *μ*m (**f** and **g**). Results were represented as mean±S.D. (*n*=3). *P<0.05; **P<0.01; ***P<0.001

To explore the biological function of miR-92b in HCC cells, first we measured the expression levels of miR-92b in five HCC cell lines and three primary human hepatocytes (PHHs) (Figure 2a). The results indicated that miR-92b expression in all five HCC cell lines were higher than that in normal hepatocytes. Interestingly, HCCLM3 cells, which possessed the highest potential of metastasis among the five HCC cell lines, expressed the highest miR-92b level. Then, miR-92b was upregulated in SMMC-7721 cells and downregulated in HCCLM3 cells. Cell Counting Kit-8 (CCK-8) assays were performed to assess the role of miR-92b in HCC cell proliferation. Compared with the control group transfected with negative control (NC) mimics, SMMC-7721 cells transfected with miR-92b mimics had a significant increase in cell viability (Figure 2b). As expected, the proliferation of HCCLM3 cells transfected with miR-92b inhibitor was slower than that of cells transfected with NC inhibitor (Figure 2c). Furthermore, we performed EdU incorporation assays and found that miR-92b could significantly increase EdU incorporation rate in HCC cells (Supplementary Figures S1a and b), and cell cycle inhibitor p21 and p27 protein levels were inhibited by miR-92b overexpression (Supplementary Figure S1c).

The wound healing and transwell assays were carried out to explore the effects of miR-92b on HCC cell metastasis. The results of wound healing assay showed that wound closure of SMMC-7721 cells with ectopic expression of miR-92b proceeded faster than that of control cells (Figure 2d), whereas suppression of miR-92b expression in HCCLM3 cells resulted in slower wound closure than that in control cells (Figure 2e). Transwell assay with Matrigel indicated that overexpression of miR-92b could promote the invasive activity of SMMC-7721 cells and knockdown of miR-92b could inhibit the invasive activity of HCCLM3 cells (Figures 2f and g). These data together suggested that miR-92b was able to promote the proliferation and metastasis of HCC cells *in vitro*.

### MiR-92b promotes HCC cell proliferation and metastasis *in vivo*

**Figure 3:**
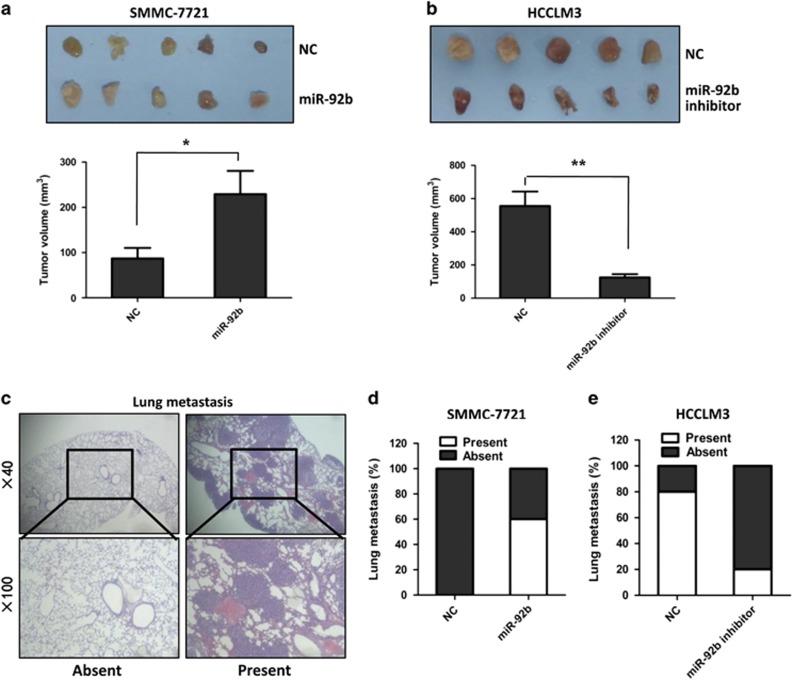
Effects of miR-92b on HCC cell proliferation and metastasis *in vivo*. (**a** and **b**) The HCC mouse model was constructed by using SMMC-7721 cells infected with miR-92b lentivirus and HCCLM3 cells infected with lentivirus-based miR-92b inhibitor. The size of local liver tumors in these two groups was calculated and compared in the diagrams. Results were represented as mean±S.D. (*n*=5). *P<0.05; **P<0.01. (**c**) Typical pictures for lung metastasis of HCC mouse model. Images were captured at × 40 (up) and × 100 (down). (**d** and **e**) The percentage of mice with or without metastatic nodules in the lungs was calculated

An HCC xenograft mouse model was used to determine the function of miR-92b *in vivo*. Overexpression and knockdown of miR-92b in the xenografted tumor cells were shown in Supplementary Figure S2. Consistent with the results *in vitro*, the tumor volumes of the miR-92b overexpression group of SMMC-7721 cells was significantly greater than that of the control group (Figure 3a). On the other hand, the volumes of tumors generated by HCCLM3 cells expressing miR-92b inhibitor were smaller than that of the control group (Figure 3b). To investigate the roles of miR-92b in HCC metastasis *in vivo*, serial sections of lung stained with hematoxylin–eosin were used to identify metastasis lesions (Figure 3c). As shown in Figure 3d, no lung metastasis was found in the control group of SMMC-7721 cells, whereas the metastasis rate reached 60% in the miR-92b group of SMMC-7721 cells. Meanwhile, the metastasis rate decreased from 80% in the control group to 20% in the miR-92b inhibitor group of HCCLM3 cells (Figure 3e). Taken together, these data suggested an important role for miR-92b in promotion of HCC growth and metastasis *in vivo*.

### MiR-92b activates the *β*-catenin signaling in HCC cells

**Figure 4:**
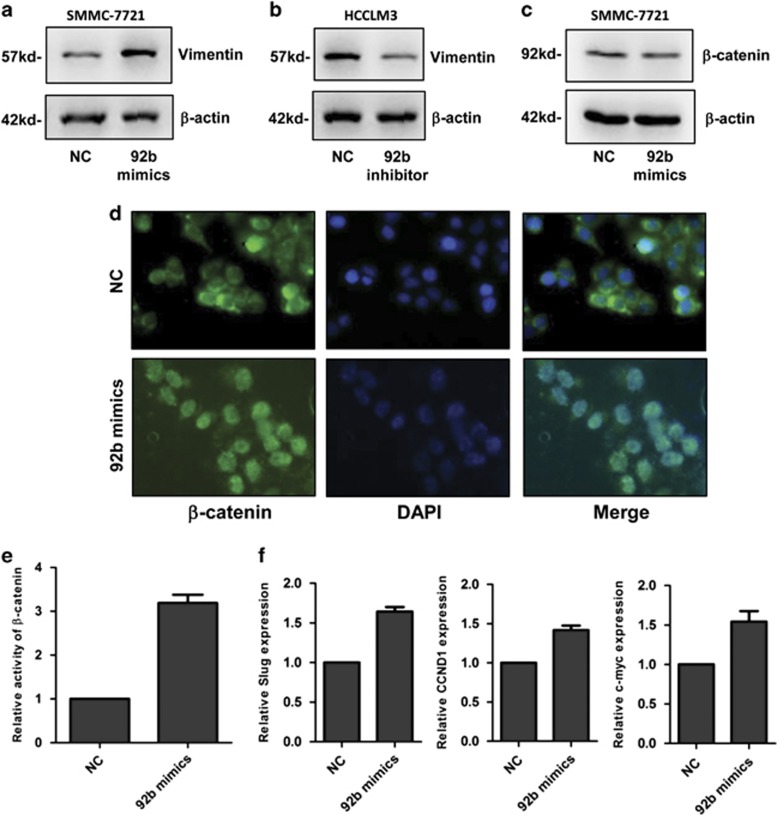
Effects of miR-92b on *β*-catenin signaling pathway in HCC cells. (**a** and **b**) Representative western blot analysis of vimentin in SMMC-7721 cells with miR-92b mimics transfection and HCCLM3 cells with miR-92b inhibitor transfection. (**c**) Western blot analysis and (**d**) immunofluorescence analysis of *β*-catenin in SMMC-7721 cells with miR-92b mimics transfection. (**e**) Reporter gene analysis of *β*-catenin transactivity in SMMC-7721 cells with miR-92b mimics transfection. Data were normalized to the activity of *β*-catenin in SMMC-7721 cells with NC mimics transfection. Results were represented as mean±S.D. (*n*=3). (**f**) qRT-PCR analysis of Slug, CCND1 and c-myc expression in SMMC-7721 cells with miR-92b mimics transfection. Data were normalized to the expression level of Slug, CCND1 or c-myc in SMMC-7721 cells transfected with NC mimics, respectively. Results were represented as mean±S.D. (*n*=3)

To explore the molecular mechanisms through which miR-92b promotes HCC tumorigenesis, we examined the expressions of multiple proteins related with cell proliferation and metastasis in SMMC-7721 cells with ectopic expression of miR-92b. The results showed that vimentin, an epithelial–mesenchymal transition-related protein, was upregulated by miR-92b (Figure 4a). Meanwhile, knockdown of miR-92b expression in HCCLM3 cells could inhibit vimentin expression (Figure 4b). Vimentin was a downstream gene of *β*-catenin, which could translocate into the nucleus to activate the transcription of target genes.^12^ Although western blot analysis showed that ectopic expression of miR-92b could not increase the expression of *β*-catenin (Figure 4c), the immunofluorescent assay indicated that overexpression of miR-92b could promote nuclear translocation of *β*-catenin in SMMC-7721 cells (Figure 4d). In addition, when *β*-catenin reporter gene was transfected together with miR-92b into SMMC-7721 cells, the activity of *β*-catenin in the cells was found to be significantly increased by miR-92b (Figure 4e). Both mRNA and protein levels of other *β*-catenin targets, including Slug, CCND1 and c-myc, were also upregulated by miR-92b (Figure 4f and Supplementary Figure S3). These results strongly suggested that miR-92b could activate the *β*-catenin signaling in HCC cells.

### Smad7 is a direct target of miR-92b

**Figure 5:**
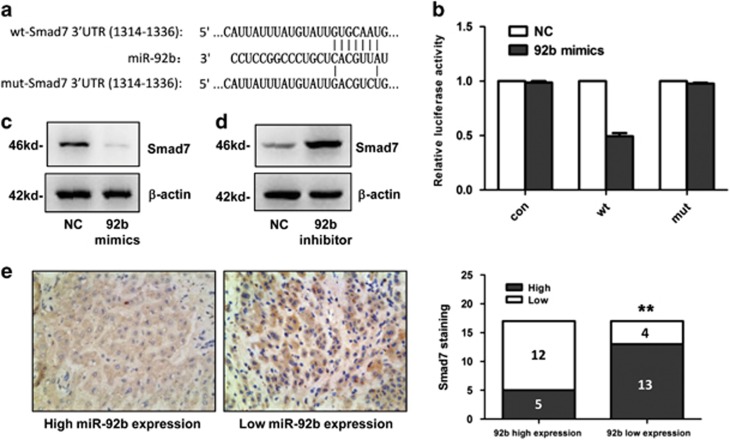
Smad7 is a direct target of miR-92b in HCC. (**a**) Diagram of the miR-92b putative binding sites and corresponding mutant sites in the 3′-UTR of Smad7 (wt, wild-type; mut, mutant type). (**b**) Effects of miR-92b on the expression of Smad7 3′-UTR-containing reporter genes. Each luciferase activity was normalized to the value obtained in the cells transfected with NC mimics. Results were represented as mean±S.D. (*n*=3). (**c** and **d**) Western blot analysis of Smad7 expression in SMMC-7721 cells with miR-92b mimics transfected and HCCLM3 cells with miR-92b inhibitor transfected. (**e**) Left Panels: representative immunohistochemistry images showing the expression of Smad7 (brown color) in HCC tissues. Right panel: a significant inverse correlation between miR-92b and Smad7 expression in HCC tissues (Spearman's *r*=−0.4714, **P<0.01)

Then, we focused on *β*-catenin signaling pathway to search for the potential target genes of miR-92b. By means of two programs including Targetscan and microRNA.org, we found that Smad7, which is known to inhibit the nucleus translocation of *β*-catenin^13^, was predicted as one of the targets for miR-92b. Interestingly, it has been reported that Smad7 was downregulated in HCC tissues compared with ANLTs,^14^ which exhibited an inverse relationship with miR-92b expression (Figure 1b). To explore if Smad7 was a direct target for miR-92b, we constructed the 3′-UTR reporter plasmids coupled with full length of Smad7 3′-UTR with wild-type (wt) or mutant (mut) miR-92b binding sites (Figure 5a). Luciferase assay showed that miR-92b could repress the expression of reporter gene containing wt 3′-UTR but not that containing mut 3′-UTR (Figure 5b). Moreover, transfection with miR-92b mimics could significantly decrease Smad7 expression in SMMC-7721 cells (Figure 5c), whereas knockdown of miR-92b could increase the Smad7 expression in HCCLM3 cells (Figure 5d). These results demonstrated that Smad7 was a direct target for miR-92b.

Furthermore, immunohistochemistry assay was performed to measure the expression levels of Smad7 in HCC tissues. According to Smad7 expression level, we divided HCC tissues into low-expression group (scores 0 and 1) and high-expression group (scores 2 and 3). The results in Figure 5e showed that miR-92b expression was inversely correlated with Smad7 expression in HCC tissues (*r*=−0.4714, *P*=0.0049). These results suggested that Smad7 might be a target of miR-92b in HCC tissues as well.

### Smad7 is essential for miR-92b-promoted HCC cell proliferation and metastasis

**Figure 6:**
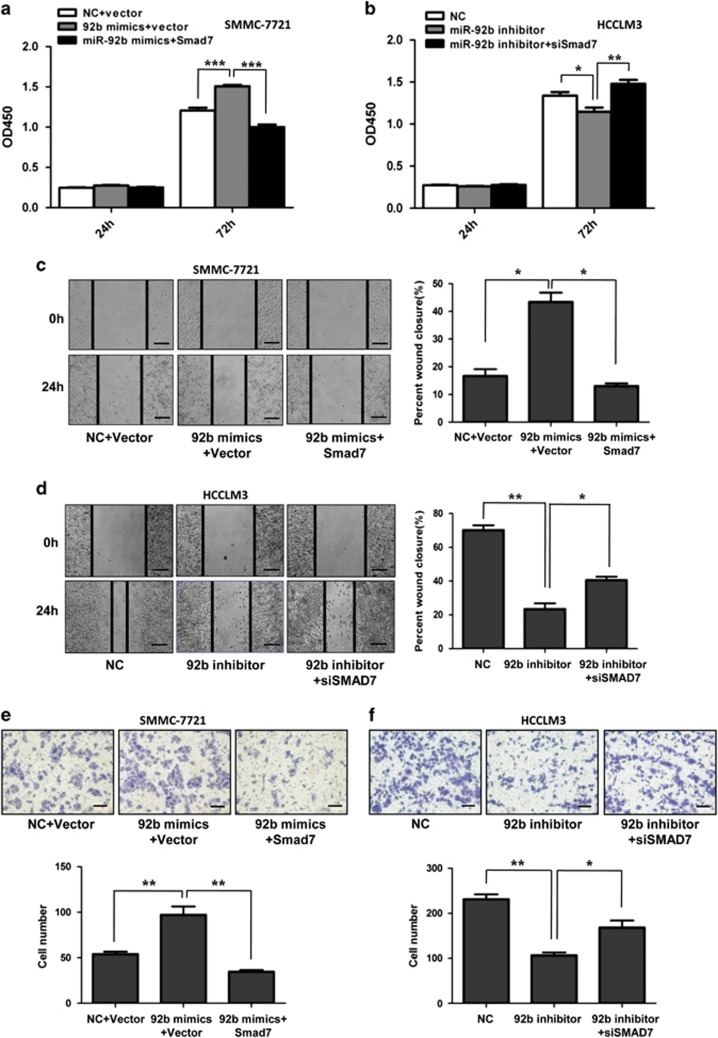
Smad7 mediates the effects of miR-92b on proliferation and metastasis of HCC cells. SMMC-7721 cells with miR-92b overexpression were transfected with Smad7-expressing plasmids, and HCCLM3 cells with miR-92b knockdown were transfected with Smad7 siRNA. (**a** and **b**) CCK-8 assays, (**c** and **d**) wound healing assays and (**e** and **f**) transwell invasion assays were performed. The percent of wound closure or the number of cells having passed through the membrane were counted and compared in the diagrams. Results were represented as mean±S.D. (*n*=3). *P<0.05; **P<0.01. Scale bars represent 500 *μ*m (**c** and **d**) and 200 *μ*m (**e** and **f**), respectively

To determine whether miR-92b-dependent promotion of HCC cell proliferation and metastasis was mediated by Smad7, gain- and loss-of-function approaches were applied. Using Smad7 expression plasmids lacking 3′-UTR, the expression of Smad7 was upregulated in miR-92b-overexpressing SMMC-7721 cells. The restoration of Smad7 significantly attenuated miR-92b-mediated promotion of proliferation (Figure 6a), wound closure (Figure 6c) and invasion (Figure 6e) of SMMC-7721 cells. In contrast, Smad7 knockdown abolished inhibition of proliferation (Figure 6b), wound closure (Figure 6d) and invasion (Figure 6f) mediated by miR-92b knockdown in HCCLM3 cells. These data further demonstrated that miR-92b could directly target Smad7 to regulate cellular proliferation and metastasis in HCC.

### LncRNA XIST and miR-92b interact with and repress each other

**Figure 7:**
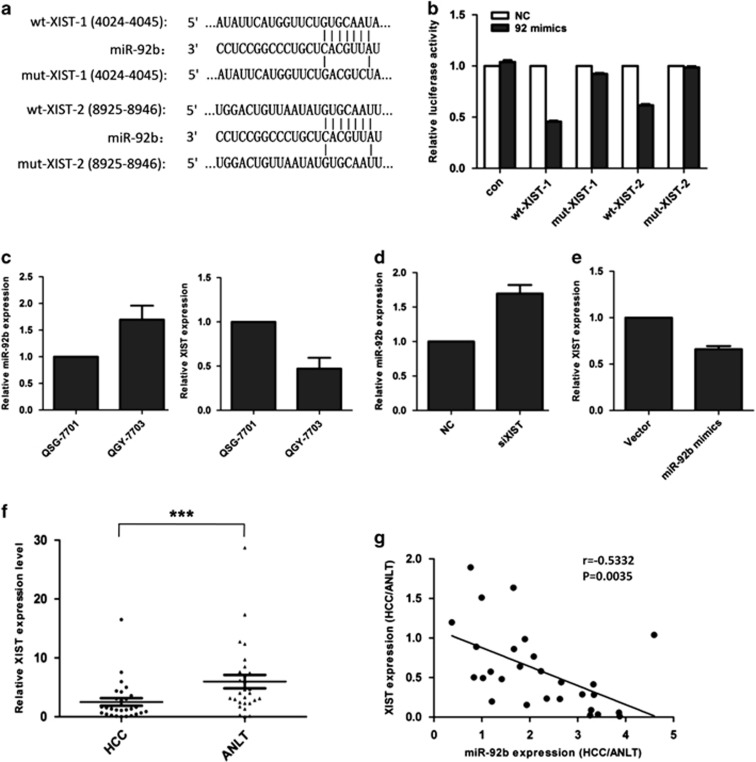
Reciprocal repression between XIST and miR-92b in HCC cells. (**a**) Diagram of the two miR-92b putative binding sites and corresponding mutant sites in XIST mRNA sequences. (**b**) Effects of miR-92b on the expression of reporter genes containing XIST-1 or XIST-2 sequences. Each luciferase activity was normalized to the value obtained in the cells transfected with NC mimics. (**c**) Relative miR-92b (left panel) or XIST expression (right panel) of QSG-7701 and QGY-7703 cells were analyzed by qRT-PCR. The data were normalized against the expression level of miR-92b or XIST in QSG-7701 cells. (**d**) Relative miR-92b expression in QGY-7703 cells transfected with XIST siRNA or NC siRNA were analyzed by qRT-PCR. The data were normalized to the expression level of miR-92b in the cells transfected with NC siRNA. (**e**) Relative XIST expression in QGY-7703 cells transfected with miR-92b or NC mimics were analyzed by qRT-PCR. The data were normalized to the expression level of XIST in the cells transfected with NC mimics. Results were represented as mean±S.D. (*n*=3). (**f**) Expression of XIST in 28 pairs of female HCC tissues and ANLTs were determined by qRT-PCR. The expression levels were compared with paired Student's *t*-test. (**g**) The negative correlation between XIST and miR-92b expression in female HCC tissues (*r*=−0.5332, ***P=0.0035, *n*=28)

In recent years, lncRNAs have been reported to act as inhibitors of miRNA expression and function.^15^ In an attempt to uncover if the expression of miR-92b was regulated by lncRNA, two bioinformatic databases including Starbase v.2.0 and miRcode were used to predict potential lncRNAs, which could interact with miR-92b. XIST, which is transcribed from the inactive X chromosome, was predicted to harbor two conserved binding sites for miR-92b. To verify any direct interaction between XIST and miR-92b, we constructed two reporter plasmids separately containing one predicted miR-92b binding site on the mRNA of XIST (wt-XIST-1 and wt-XIST-2), and two corresponding reporter plasmids with mutant miR-92b binding sites (mut-XIST-1 and mut-XIST-2) (Figure 7a). Luciferase reporter gene assay showed that miR-92b could significantly inhibit the reporter activities of both wt-XIST-1 and wt-XIST-2, but not mut-XIST-1 nor mut-XIST-2 (Figure 7b). This indicated that miR-92b probably interacted with XIST. Interestingly, XIST could hardly be detected in the male HCC tissues or male cell lines including SMMC-7721 or HCCLM3. We subsequently detected the expression of XIST and miR-92b in female HCC cell line QGY-7703 and female normal liver cell line QSG-7701. The results showed that the level of XIST was higher in QSG-7701 cells than that in QGY-7703 cells, exhibiting an inverse relationship with the miR-92b expressions in the two cell lines (Figure 7c). The results of qRT-PCR indicated that miR-92b expression was upregulated after knockdown of XIST in QGY-7703 cells (Figure 7d), whereas the XIST level could also be repressed by miR-92b overexpression (Figure 7e). These results suggested that there was reciprocal repression between miR-92b and XIST.

Furthermore, we detected XIST expression in female HCC patients. The result showed that the expression of XIST in HCC tissues was much lower than that in corresponding ANLTs (Figure 7f). Similarly, the correlation between miR-92b and XIST expression in HCC tissues was also inverse (Figure 7g).

### XIST affects HCC cell proliferation and metastasis by regulating miR-92b expression

**Figure 8:**
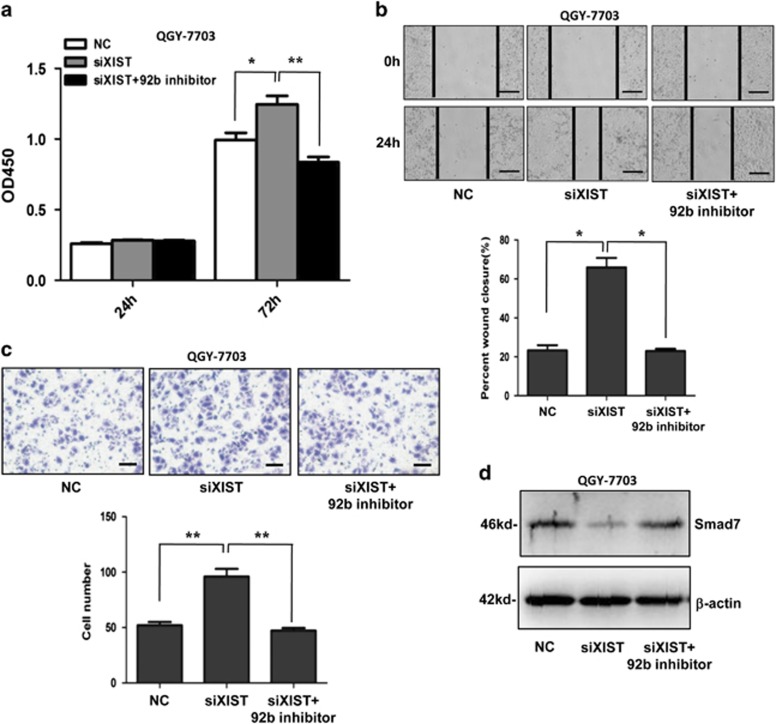
Effects of XIST and miR-92b knockdown on proliferation and metastasis of HCC cells. QGY-7703 cells were co-transfected with siXIST plus miR-92b inhibitor or NC inhibitor. (**a**) CCK-8 assays, (**b**) wound healing assays, (**c**) transwell invasion assays and (**d**) western blot assays for Smad7 were performed. The percent of wound closure and the number of cells that passed through the membrane were counted and compared in the diagrams. Results were represented as mean±S.D. (*n*=3). *P<0.05; **P<0.01. Scale bars represent 500 *μ*m (**b**) and 200 *μ*m (**c**), respectively

Then the question was raised: could XIST affect the cellular functions of HCC cells by regulating the expression of miR-92b? To answer it, we knocked down XIST expression with siRNA (siXIST) in QGY-7703 cells. The results showed that the proliferation (Figure 8a), migration (Figure 8b) and invasion (Figure 8c) of cells with siXIST were significantly enhanced compared with that of control cells. Meanwhile, knockdown of miR-92b in QGY-7703 cells could abolish the enhancing effects of siXIST on cell proliferation and metastasis (Figures 8a–c). Interestingly, Smad7 expression was inhibited by siXIST and the inhibitory effect was abolished by miR-92b inhibitor (Figure 8d), which validated the XIST/miR-92b/Smad7 signaling pathway. These results indicated that XIST could affect tumor cell proliferation and metastasis of HCC by regulating the expression of miR-92b, indeed.

## Discussion

More and more evidences have indicated that the deregulation of miRNAs might be involved in progression of many cancers including HCC.^16^ miRNA expression profiling has been used as an approach for identification of molecular biomarkers for tumor occurrence, classification and prognosis.^17, 18^ In this study, we found that miR-92b was upregulated in HCC tissues compared with that in ANLTs. Moreover, miR-92b expression was significantly correlated with gender and microvascular invasion of HCC. These data suggested that miR-92b might participate in the tumorigenesis, gender difference and metastasis of HCC. Plasma miRNAs have been reported to be diagnostic and prognostic biomarkers for many diseases.^19, 20^ Our results showed that plasma miR-92b level was significantly higher in HCC patients than that in patients without liver diseases. Circulating AFP has been a useful biomarker for HCC, and the significant positive correlation between plasma AFP and miR-92b strongly suggested that circulating miR-92b might be another potential biomarker for diagnosis of HCC.

It has been reported that miR-92b could participate in the tumorigenesis of glioma and non-small-cell lung cancer,^21, 22^ but until now little has been known about the role and molecular mechanism of miR-92b in disease progression of HCC. In this study, we found that miR-92b could affect *β*-catenin signaling and promote HCC cell proliferation and metastasis *in vitro* and *in vivo*, which revealed the crucial roles of miR-92b in HCC. It has been reported that miR-92b could target Dickkopf-3 (DKK3) and Nemo-like kinase (NLK) to regulate Wnt/*β*-catenin signaling in glioma cells.^23, 24^ Unfortunately, there was no significant downregulation of DKK3 and NLK in HCC tissues compared with ANLTs (data not shown), which was consistent with the results from previous reports.^25, 26, 27^ Neither DKK3 nor NLK had the inverse relationship with miR-92b expression in HCC tissues, implying DKK3 and NLK might not be key downstream targets of miR-92b in HCC progression. In view of the fact that one miRNA might downregulate large numbers of target genes by binding to complementary sequences in the 3′-UTR of target mRNAs, the downstream targets of a miRNA in one particular tissue or cell type should be determined by the gene expression patterns specific to the tissue or cell. One previous study has reported that Smad7, a potential inhibitor of TGF-*β*/*β*-catenin signaling pathway, was significantly downregulated in HCC tissues.^14^ Consistently, our results also showed that Smad7 was a direct target of miR-92b and exhibited an inverse correlation with miR-92b expression in HCC tissues. Moreover, the ectopic expression of Smad7 could significantly attenuate miR-92b-induced cell proliferation and metastasis, which further demonstrated the key roles of miR-92b-depressed Smad7 expression in HCC progression.

Furthermore, we also showed the evidence for the reciprocal regulation between miR-92b and XIST. XIST has been reported to exert important effects on many tumors including testicular germ cell tumors, ovarian cancer, hematologic cancer and glioblastoma.^11, 28, 29, 30^ In glioblastoma, XIST could promote tumor cell proliferation and invasion.^11^ While XIST was reported to be a potent suppressor of hematologic cancer.^30^ In different tumors, it seems that XIST could have different roles as an oncogene or a tumor suppressor. Our results showed for the first time that knockdown of XIST could upregulate the expression of miR-92b to promote HCC cell proliferation and metastasis. Therefore, the reciprocal regulation of miR-92b and XIST might have an important role in HCC progression.

Previous studies had shown that XIST was expressed mainly in female cells and induced the silencing of X chromosome.^31^ It was reported that the incidence rate of HCC in males was two to three times higher than that in females.^32^ It has been known that the gender disparity of HCC occurrence was related with many factors including sex hormones and cytokines.^33^ However, the role of gender-specific genes in HCC tumorigenesis has been rarely reported. In this study, we found that XIST was expressed mainly in female but not in male HCC tissues and its expression level in female HCC tissues was significantly lower than that in ANLTs. In view of the inhibitory effects of XIST in HCC progression, the interaction between XIST and miR-92b might partially interpret why females suffer less HCC than males. In the future, our work will focus on the correlation between gender disparity of HCC incidence and different expression patterns of XIST. Animal models such as nude mice would also be selected to further demonstrate this discovery in this study.

In summary, our results showed for the first time that miR-92b was upregulated in HCC tissue and plasma. Increased expression of miR-92b could promote HCC cell proliferation and metastasis *in vitro* and *in vivo*. Smad7 was one of the direct targets of miR-92b and participated in the miR-92b-involved tumorigenesis of HCC via *β*-catenin signaling. LncRNA XIST could suppress miR-92b expression to inhibit the proliferation and metastasis of HCC cells. Our study not only revealed the important role of XIST/miR-92b/Smad7 signaling pathway in HCC pathogenesis (Supplementary Figure S4) but also implicated the potential role of both miR-92b and XIST in the clinical diagnosis and treatment of HCC.

## Materials and Methods

### Tissue and plasma specimens of patients

Forty-eight pairs of HCC tissues and ANLTs were randomly selected from patients who underwent liver resection at the Department of Hepatobiliary and Pancreatic Surgery, Affiliated Hospital of Qingdao University during January 2013 to December 2015. Histopathology was evaluated independently by two certified pathologists. The clinicopathological data are shown in Table 1. Plasma samples from 31 patients with HCC and 29 patients without liver diseases were collected for RNA extraction. The clinicopathological data are shown in Supplementary Table S1. The study protocol conformed to the Ethical Guidelines of the 1975 Declaration of Helsinki, revised in 2000. All human materials were obtained with informed consent and approved by the Ethics Committee of Affiliated Hospital of Qingdao University.

### Cell culture

In this study, one normal liver cell line (QSG-7701) and six HCC cell lines (SMMC-7721, Huh-6, Huh-7, HepG2, HCCLM3 and QGY-7703) were used. All the cell lines were obtained from Cell Resource Center of Shanghai Institutes for Biological Sciences, Chinese Academy of Sciences (Shanghai, China) and cultured in DMEM medium containing 10% fetal bovine serum (Gibco, Grand Island, NY, USA) in a humidified atmosphere of 5% CO_2_ at 37 °C. PHHs were isolated from normal liver tissues far away from liver tumor tissues of patients undergoing liver resection. The isolation and culture of primary human hepatocytes were conducted as described previously.^34^

### Quantitative reverse transcription-PCR

Total RNAs of tissue or plasma were extracted using Trizol (Invitrogen, Carlsbad, CA, USA) according to the instructions provided by the manufacturer. For the extraction of circulating RNA from plasma, SV40 miRNA mimic (Qiagen, Valencia, CA, USA) 1 pmol/200 *μ*l plasma was added as spike-in RNA for later normalization. After reverse transcription, cDNA was amplified by using SYBR-Green Premix (Takara, Otsu, Japan). The expression of miR-92b and XIST in tissue was, respectively, normalized to the expression of U6 and GAPDH. The data were analyzed by delta Ct method. The primers used in this study were listed in Supplementary Table S2.

### Small RNA sequencing

Total RNAs from eight HCC tissue samples and eight ANLT samples were, respectively, pooled and sent to Beijing Genomics Institute (Shenzhen, China) for sequencing of small RNAs. The sequencing analysis was performed on Illumina HiSeq2000 platform (Illumina Inc., San Diego, CA, USA). The miRNA expression profile was sorted using Cluster 3.0 software (University of Tokyo, Human Genome Center).

### Plasmids and luciferase reporter assay

Smad7-expressing plasmid was obtained from OriGene Technologies (Rockville, MD, USA). To generate reporter construct, one fragment of Smad7 3′-UTR including putative miR-92b complementary sequences and two fragments of XIST mRNA containing predicted miR-92b binding site 1 or 2 were separately fused to a modified pcDNA3.1 vector containing a luciferase gene, which was inserted upstream of cloning sites. Mutant reporter plasmids were prepared by Mutagenesis Kit (Stratagene, La Jolla, CA, USA). TOP/FLASH reporter gene including *β*-catenin binding sites was obtained from Millipore (Billerica, MA, USA). The reporter constructs were transfected into cells, which were subsequently lysed for reporter assays. Luciferase activity assay was conducted using Dual Luciferase Assay System (Promega, Madison, WI, USA). pRL-TK plasmid (Promega) was used to normalize the transfection efficiency.

### Oligonucleotide and plasmid transfection

Oligonucleotides including miR-92b mimics, miR-92b inhibitor, Smad7 siRNA, XIST siRNA and NC oligonucleotides were purchased from GenePharma (Shanghai, China). The sequences of effective Smad7 siRNA were described previously.^35^ The sequences and efficiency of three XIST siRNAs were showed in Supplementary Figure S5, and siXIST-1 was selected for subsequent study because of its highest effectivity. Oligonucleotides were transfected into cells by Hiperfect transfection reagent (Qiagen), and the transfection of plasmids were conducted using Lipofectamine 3000 (Invitrogen, Carlsbad, CA, USA).

### Cell proliferation assays

Cell proliferation was measured by the CCK-8 (Dojindo, Kumamoto, Japan). Briefly, cells were seeded into a 96-well plate and cultured at 37 °C for 24 h before transfection. Then, cells were transfectd with corresponding oligonucleotides or plasmids. At various time points, 10 *μ*l CCK-8 solution was added into each well of the plate. The plates were incubated at 37 °C for 1 h, and the absorbance at 450 nm was measured.

### Wound-healing assays

For wound healing assays, cells were plated into a 12-well plate and cultured at 37 °C for 24 h. Wounds were created in monolayers of cells using a 10 *μ*l pipette tip. Cells were washed to remove cellular debris and incubated in DMEM without FBS at 37 °C. Images were taken at different points of time following wounding. The wound area was measured and the percentage of the wound healing was calculated by Image J software (NIH, Bethesda, MD, USA).

### Transwell invasion assays

Cell invasion assays were performed using 24-well transwell chambers with 8.0 *μ*m pore size polycarbonate membrane (Corning Incorporated, Corning, NY, USA). Cells were seeded on the top side of the membrane precoated with Matrigel (BD, Franklin Lakes, NJ, USA). After incubation, cells inside the upper chamber were removed with cottons swabs. Invaded cells on the lower membrane surface were fixed and then stained with 5% crystal violet.

### Western blot

Total protein was extracted and separated by sodium dodecyl sulfate-polyacrylamide gel electrophoresis and then transferred onto 0.45 *μ*m PVDF membrane (Millipore). The following primary antibodies were used in the immunoblotting assays: antibodies for Smad7 (sc-101152; Santa Cruz Biotechnology, Santa Cruz, CA, USA), vimentin (no. 3390; Cell Signaling Technology, Danvers, MA, USA), *β*-actin (no. 3700; Cell Signaling Technology), CCND1 (no. 2926; Cell Signaling Technology), c-myc (ab17356; Abcam, Cambridge, MA, USA), Slug (no. 9585; Cell Signaling Technology), P21 (sc-6246; Santa Cruz), P27 (no. 3688; Cell Signaling Technology) and *β*-catenin (no. 8480; Cell Signaling Technology).

### Immunohistochemistry

Immunohistochemistry was performed on paraformaldehyde-fixed paraffin sections. Smad7 antibody (sc-11392; Santa Cruz) was used in immunohistochemistry with streptavidin peroxidase-conjugated method. The percentage of positive tumor cells was graded as per the following criteria: 0, <10% 1, 10–30% 2, 31–50% 3, >50%. Patients with different Smad7 expression in HCC tissues were divided into the low-expression group (0 or 1) and the high-expression group (2 or 3).

### Immunofluorescence

Cells cultured on glass coverslips were fixed with 4% paraformaldehyde, blocked with 5% bovine serum albumin and incubated with *β*-catenin antibody (no. 8480; Cell Signaling Technology) overnight at 4 °C. Then, cells were rinsed three times and incubated with FITC-conjugated goat anti-rabbit IgG (F0382; Sigma, Steinheim, Germany) for 2 h at room temperature. Coverslips were mounted using ProLong Gold antifade reagent with DAPI (Invitrogen) and imaged using a LEICA DMI4000B microscope (Leica, Heidelberg, Germany). Image analysis was performed by using the Image J software.

### HCC xenograft mouse model

MiR-92b overexpression and knockdown lentivirus as well as the NC lentivirus were purchased from GenePharma. After infection, HCC cells (5 × 10^6^) were suspended in 100 *μ*l phosphate-buffered saline and injected subcutaneously to the posterior flank of the BALB/c nude mice purchased from the Shanghai Institute of Materia Medica (Shanghai, China). One week later, the subcutaneous tumor tissues were removed and implanted into the liver of nude mice. After 5 weeks of implantation, the mice were killed and the tumor was separated from normal liver tissue. The tumor volumes were measured and the size was calculated as follows: tumor volume (mm^3^)=(length × width^2^)/2. The lung of mice were fixed with formaldehyde solution and embedded with paraffin. The paraffin-fixed tissues were serial sectioned and stained with hematoxylin–eosin to identify metastastic lesions. All animal studies were conducted in the Animal Institute of Qingdao University according to the protocols approved by the Medical Experimental Animal Care Commission of Qingdao University.

### Statistical analysis

Statistical analysis was performed using the SPSS program (version 18.0; SPSS, Chicago, IL, USA). Data were presented as mean±S.D. Statistical significance was calculated by Student's *t*-test, *χ*^2^ test, Fisher's exact test or one-way ANOVA. Pearson's or Spearman's analysis was used in correlation analysis. *P*<0.05 was considered as statistically significant.

## Figures and Tables

**Table 1:** Correlation between the clinicopathologic characteristics and miR-92b expression in HCC tissues

	**Total no. of**	**No. of patients**		
	**patients,** ***n*****=48**	**miR-92b**^**high**^	**miR-92b**^**low**^	***P*****-value**
*Gender*				
Male	20	11	9	
Female	28	23	5	**0.041**
				
*Age (years)*				
⩽60	31	23	8	
>60	17	11	6	0.489
				
*Tumor size (cm)*				
⩽5	33	22	11	
>5	15	12	3	0.549
				
*AFP (ng/ml)*				
⩽20	18	11	7	
>20	30	23	7	0.251
				
*Hepatitis B status*				
Negative	7	4	3	
Positive	41	30	11	0.680
				
*Liver cirrhosis*				
Absent	12	10	2	
Present	36	24	12	0.463
				
*Number of tumors*				
1	40	29	11	
⩾2	8	5	3	0.887
				
*Microvascular invasion*				
Absent	24	13	11	
Present	24	21	3	**0.026**
				
*Liver capsule invasion*				
No	16	11	5	
Yes	32	23	9	0.822
				
*TNM stage*				
I	39	28	11	
II–III	9	6	3	0.760
				
*BCLC stage*				
0–A	38	28	10	
B–C	10	6	4	0.648

Abbreviations: AFP, *α*-fetoprotein; ANLT, adjacent non-tumor liver tissue; BCLC, Barcelona Clinic Liver Cancer; HCC, hepatocellular carcinoma; TNM, tumor node metastasis

miR-92b^high^ or miR-92b^low^: miR-92b expression in HCC tissues was higher or lower than that in corresponding ANLTs. Bold numbers indicate significant differences (*P*<0.05)

## Abbreviations

HCC: hepatocellular carcinoma
miRNA: microRNA
lncRNA: long non-coding RNA
3′-UTR: 3′-untranslated region
mRNA: messenger RNA
XIST: X-inactive specific transcript
ANLT: adjacent non-tumorous liver tissue
qRT-PCR: quantitative reverse transcription-PCR
PHH: primary human hepatocyte
NC: negative control
AFP: *α*-fetoprotein
wt: wild type
mut: mutant
DKK3: Dickkopf-3
NLK: Nemo-like kinase
CCK-8: Cell Counting Kit-8
