# Supplementary material for: MicroRNA-92b promotes hepatocellular carcinoma progression by targeting Smad7 and is mediated by long non-coding RNA XIST
Source: Cell Death Dis. 2016 Apr 21;7(4):e2203–. doi: 10.1038/cddis.2016.100 (PMC4855645; doi:10.1038/cddis.2016.100)
Supplement: Supplementary Information [file cddis2016100x1.docx]

**Supplementary Materials and Methods**

**EdU incorporation assay.** EdU incorporation assay was performed using Click-iT EdU HCS assay kit (Invitrogen; Carlsbad, CA, USA). Briefly, cells were seeded in 24-well plates, transfected with corresponding oligonucleotides for 48h and followed by EdU incubation. EdU were detected by fluorescent-azide coupling reaction and the cells were stained with HCS NuclearMask™ Blue. Images were visualized using a LEICA DMI4000B microscope.

**Supplementary Figure Legends**

**Figure S1.** Effects of miR-92b on HCC cell proliferation. **(a-b)** EdU incorporation assays were performed in SMMC-7721 cells transfected with miR-92b mimics **(a)** and HCCLM3 cells with miR-92b inhibitor transfection **(b)**. Representative images were shown and the EdU incorporation rate was expressed as the ratio of EdU positive cells to total nuclear stain positive cells. Results were represented as mean ± S.D. (n = 3). **P* < 0.05. **(c)** Western blot analysis of p21 and p27 expression in SMMC-7721 cells with miR-92b or NC mimics transfection.

**Figure S2.** qRT-PCR analysis of miR-92b expression in xenograft tumors generated by SMMC-7721 **(a)** or HCCLM3 cells **(b)**. Data were normalized to U6 expression and the expression levels were compared with unpaired Student‘s *t* test. Results were represented as mean ± S.D. (n = 5). ***P* < 0.01; ****P* < 0.001.

**Figure S3.** Western blot analysis of Slug, CCND1 and c-myc expression in SMMC-7721 cells with miR-92b or NC mimics transfection.

**Figure S4.** Summary diagram of the XIST/miR-92b/Smad7 pathway in HCC. In HCC cells, miR-92b is highly expressed. One of the miR-92b targets is Smad7 which inhibits the nuclear translocation and transactivity of β-catenin. The expression of Smad7 is repressed by miR-92b which could bind to the 3’UTR of Smad7 mRNA. LncRNA XIST which exists in female hepatocytes interacts with miR-92b and suppresses its expression and function.

**Figure S5.** Sequences and inhibitory efficiency of three XIST siRNAs. **(a)** The sequences of three XIST siRNAs. **(b)** Relative mRNA expression of XIST in QGY-7703 cells transfected with NC and three XIST siRNAs. The data were normalized to the expression level of XIST in the cells transfected with NC siRNA. Results were represented as mean ± S.D. (n = 3).
