# Supplementary figures and images for: MicroRNA-92b promotes hepatocellular carcinoma progression by targeting Smad7 and is mediated by long non-coding RNA XIST
Source: Cell Death Dis. 2016 Apr 21;7(4):e2203–. doi: 10.1038/cddis.2016.100 (PMC4855645; doi:10.1038/cddis.2016.100)

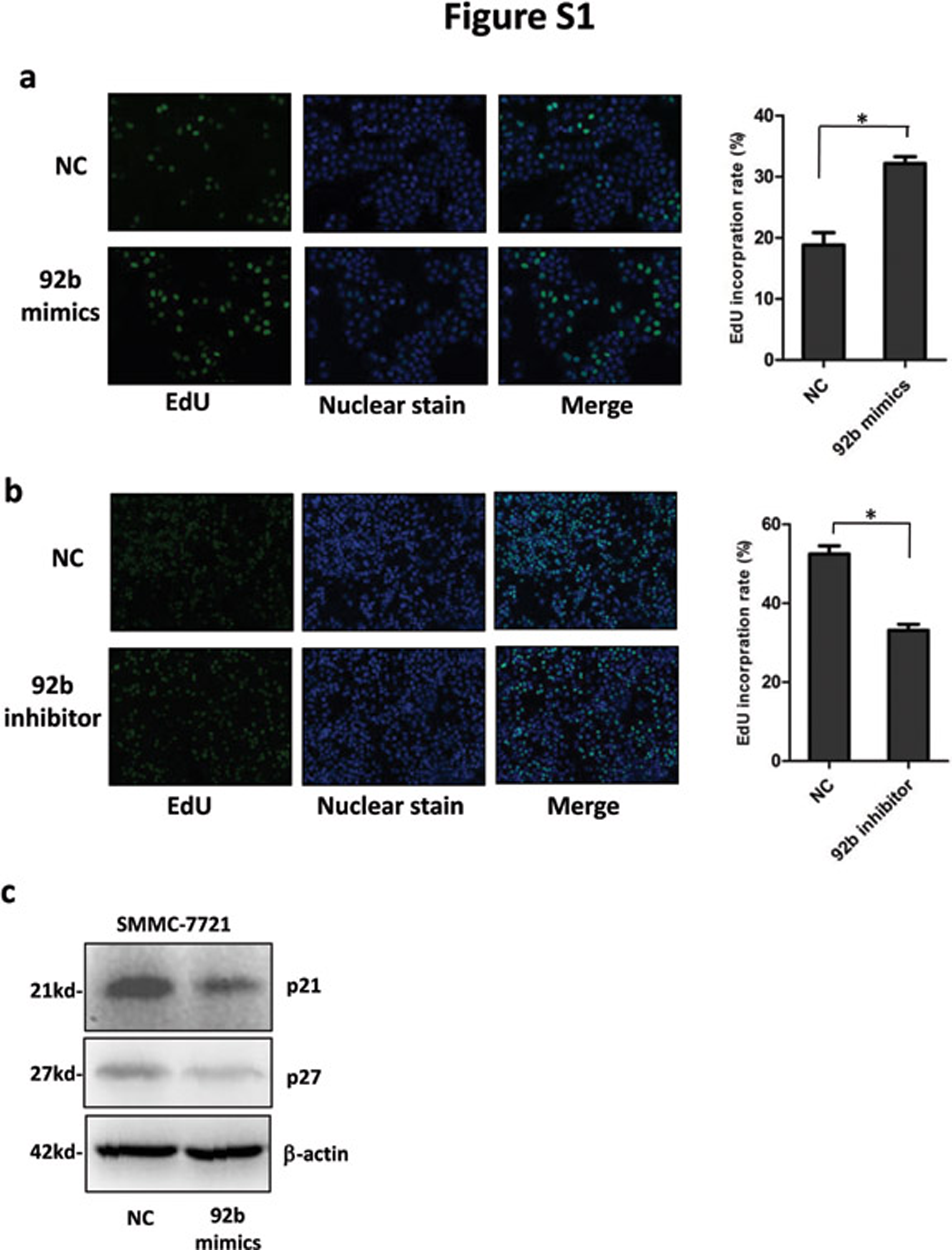

Supplement: Supplementary Figure S1 [file cddis2016100x2.tif]

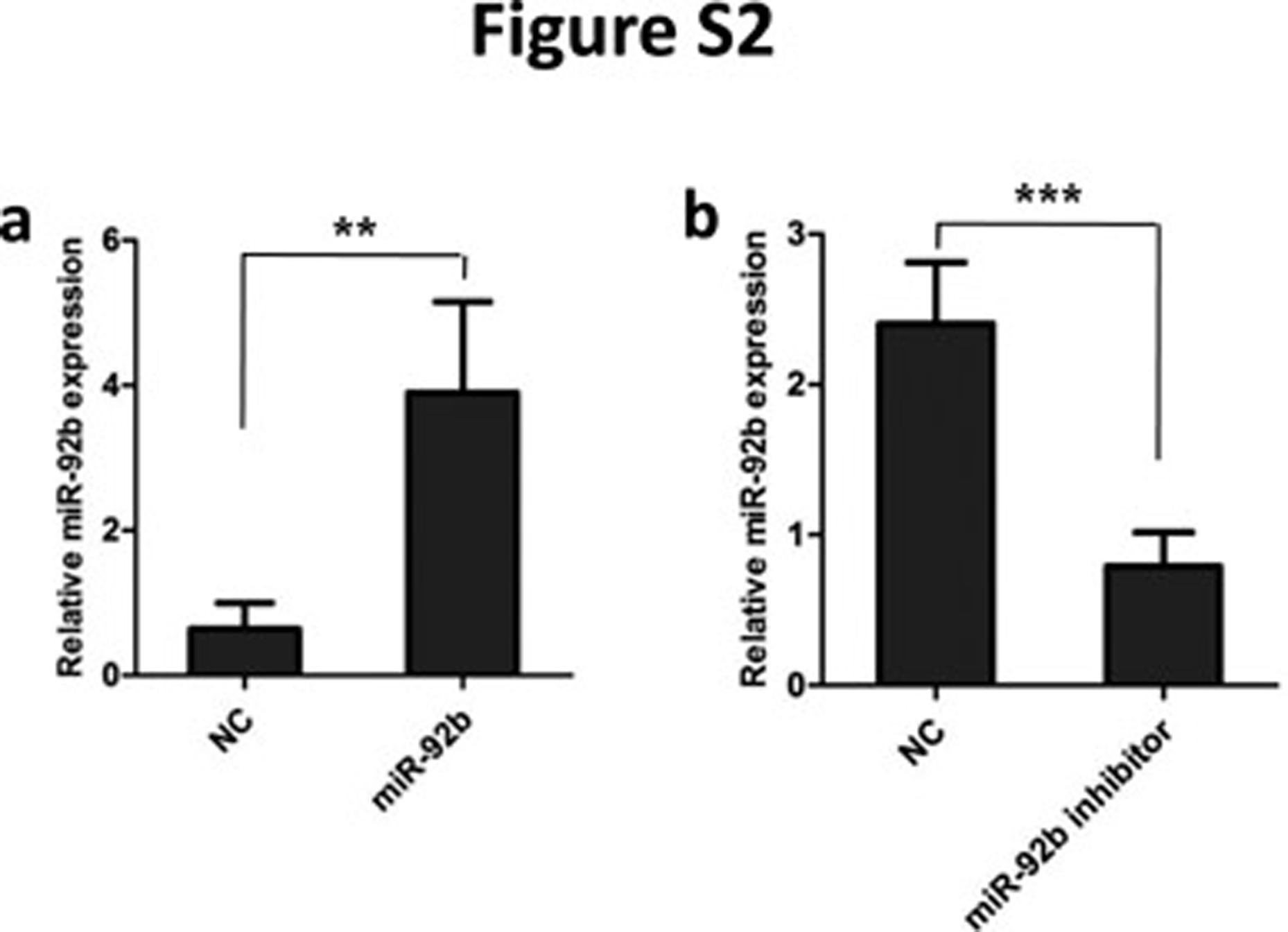

Supplement: Supplementary Figure S2 [file cddis2016100x3.tif]

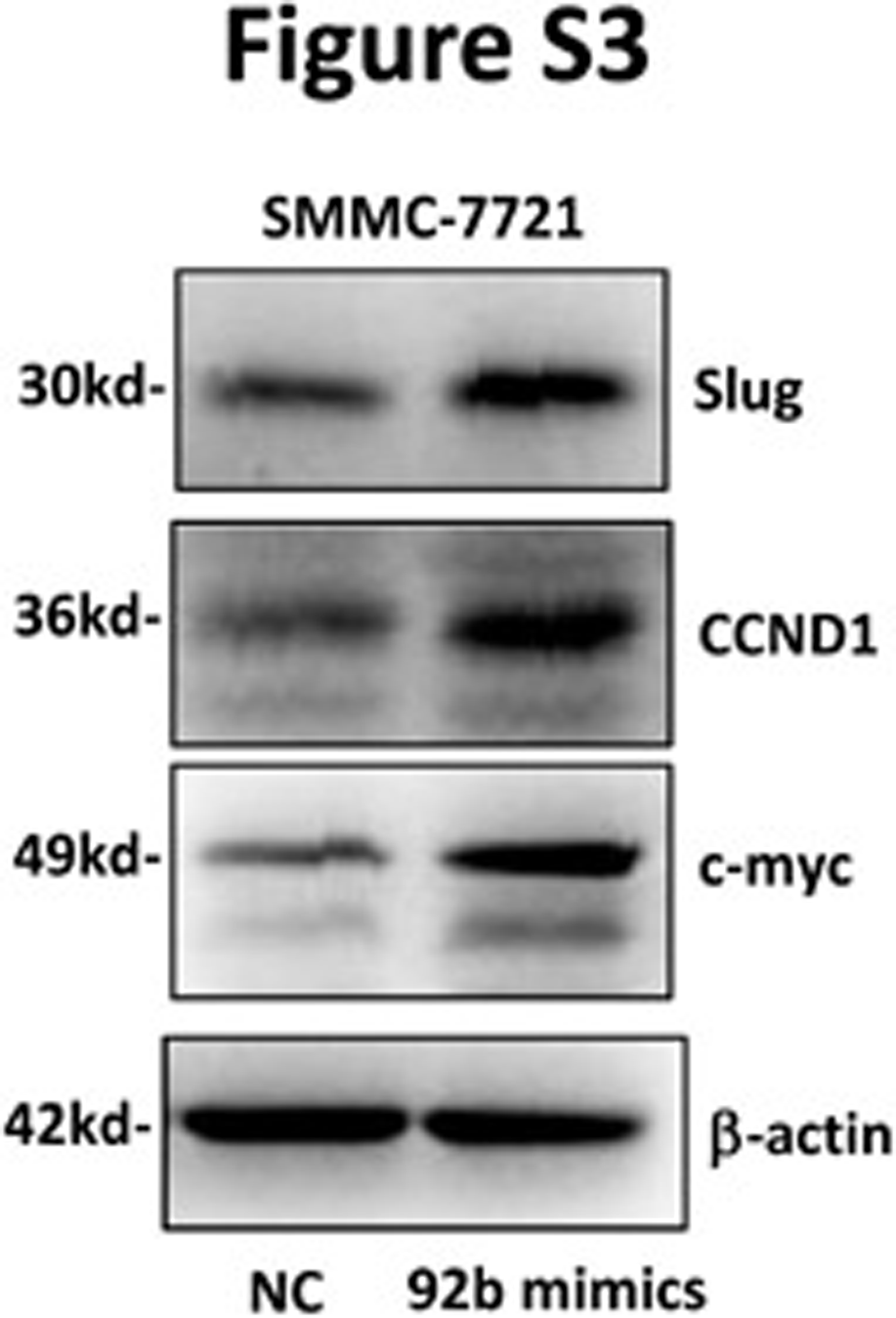

Supplement: Supplementary Figure S3 [file cddis2016100x4.tif]

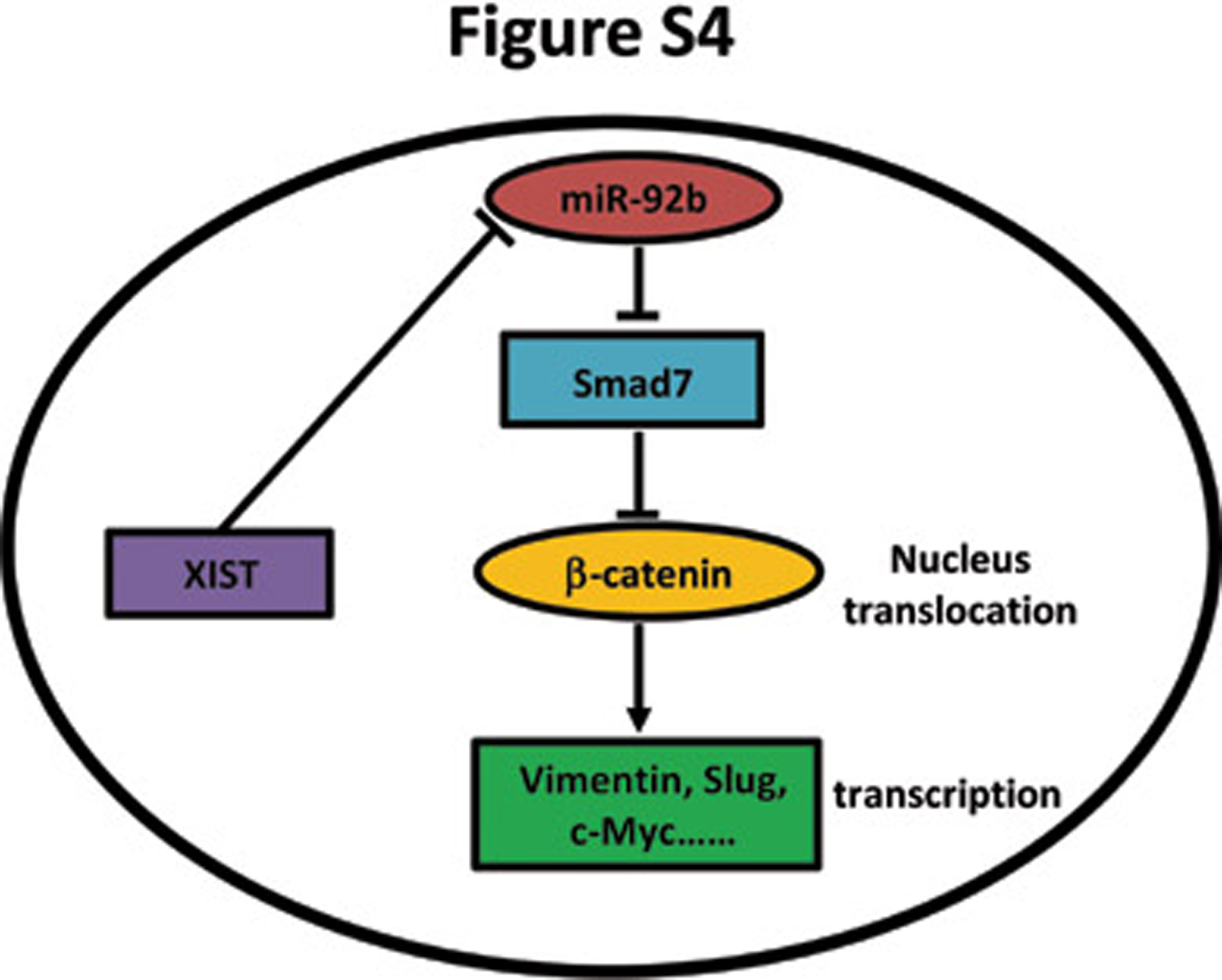

Supplement: Supplementary Figure S4 [file cddis2016100x5.tif]

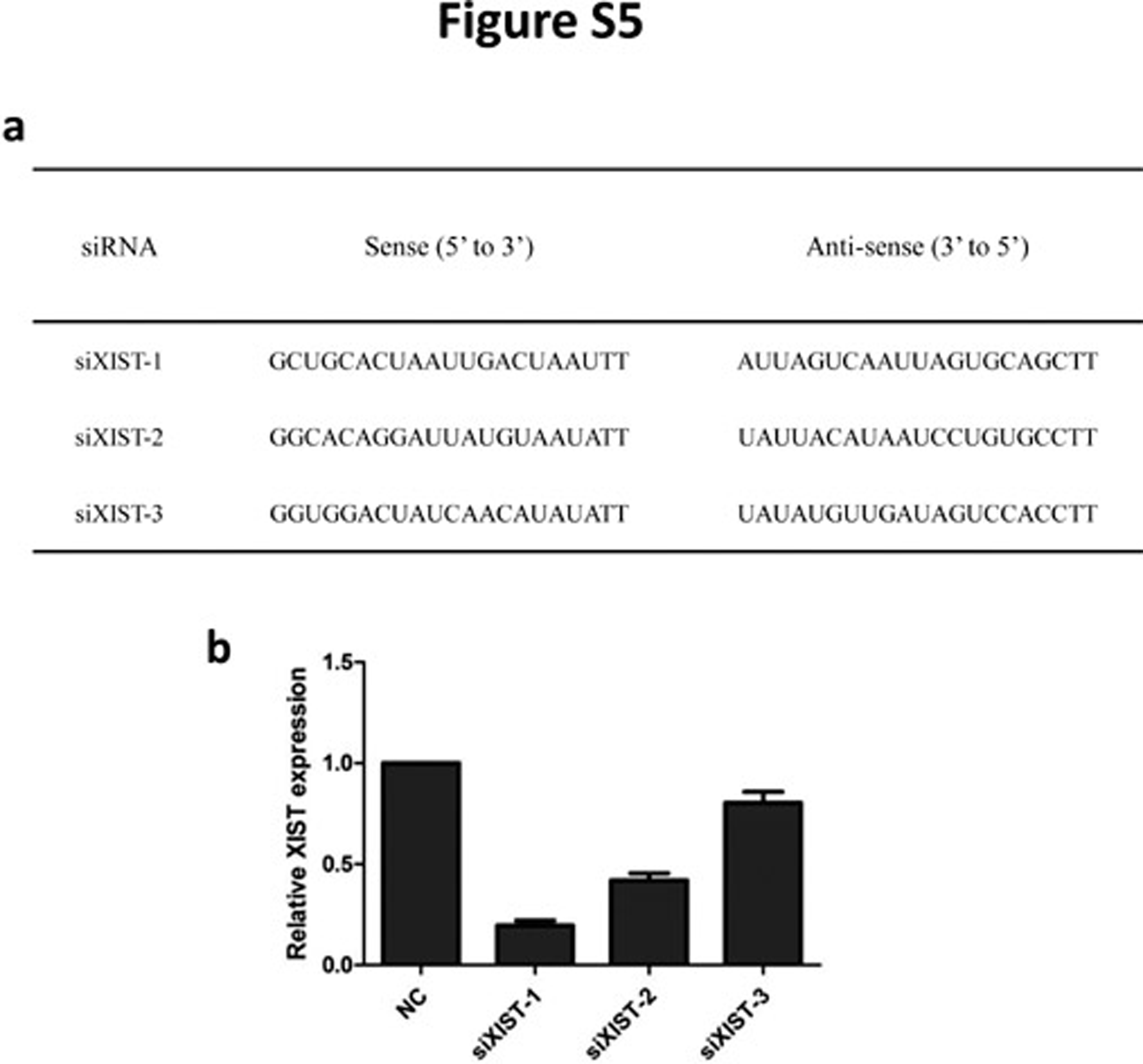

Supplement: Supplementary Figure S5 [file cddis2016100x6.tif]
