# Supplementary material for: MicroRNA-92b promotes hepatocellular carcinoma progression by targeting Smad7 and is mediated by long non-coding RNA XIST
Source: Cell Death Dis. 2016 Apr 21;7(4):e2203–. doi: 10.1038/cddis.2016.100 (PMC4855645; doi:10.1038/cddis.2016.100)
Supplement: Supplementary Table S1 [file cddis2016100x7.docx]

**Table S1.** Clinical characteristics of 31 HCC patients and 29 non-HCC patients.

| Factors | HCC patients  (n = 31) | Non-HCC patients  (n=29) | *P*-value |
| --- | --- | --- | --- |
| Gender(male/female) | 19/12 | 18/11 | 0.951 |
| Age (years)^*^ | 55.7±10.7 | 55.9±10.9 | 0.983 |
| AFP (ng/mL)^*^ | 355.7±601.5 | 3.2±1.6 | 0.000 |
| Tumor size (cm)^*^ | 4.6±2.5 |  |  |
| HBsAg (negative/positive) | 3/28 |  |  |
| Liver cirrhosis (no/yes) | 6/25 |  |  |
| Number of tumors (single/multiple) | 28/3 |  |  |
| Microvascular invasion (absent/present) | 17/14 |  |  |
| Liver capsule invasion (no/yes) | 7/24 |  |  |
| TNM stage (I/ II-III) | 27/4 |  |  |
| BCLC stage ( 0-A/ B-C) | 26/5 |  |  |

^*^Data are mean ± SD.
