# Supplementary material for: MicroRNA-92b promotes hepatocellular carcinoma progression by targeting Smad7 and is mediated by long non-coding RNA XIST
Source: Cell Death Dis. 2016 Apr 21;7(4):e2203–. doi: 10.1038/cddis.2016.100 (PMC4855645; doi:10.1038/cddis.2016.100)
Supplement: Supplementary Table S2 [file cddis2016100x8.docx]

**Table S2.** Primers used in this study.

| **Primer** | **Sequences** |
| --- | --- |
| miR-92b RT-Primer | GTCGTATCCAGTGCAGGGTCCGAGGTATTCGCACTGGATACGACGGAGGCCGG |
| miR-92b Forward primer | TATATTGCACTCGTCCCGGC |
| miR-92b Reverse Primer | CTAGTGCAGGGTCCGAGGTATT |
| c-Myc Forward primer | CAAGAGGCGAACACACAACGT |
| c-Myc Reverse primer | GGGCCTTTTCATTGTTTTCCA |
| CCND1 Forward primer | CTGGAGGTCTGCGAGGAACA |
| CCND1 Reverse primer | CTTAGAGGCCACGAACATGCA |
| XIST Forward primer | CGGGTCTCTTCAAGGACATTTAGCC |
| XIST Reverse primer | GCACCAATACAGAGGAATGGAGGG |
| Slug Forward primer | CATGCCTGTCATACCACAAC |
| Slug Reverse primer | GGTGTCAGATGGAGGAGGG |
| GAPDH Forward primer | GAAGATGGTGATGGGATTTC |
| GAPDH Reverse primer | GAAGGTGAAGGTCGGAGT |
| U6 Forward primer | TCGCTTCGGCAGCACATA |
| U6 Reverse primer | TTTGCGTGTCATCCTTGC |
